# Near Infrared Responsive Gold Nanorods Attenuate Osteoarthritis Progression by Targeting TRPV1

**DOI:** 10.1002/advs.202307683

**Published:** 2024-02-15

**Authors:** Weitong Li, Zhongyang Lv, Peng Wang, Ya Xie, Wei Sun, Hu Guo, Xiaoyu Jin, Yuan Liu, Ruiyang Jiang, Yuxiang Fei, Guihua Tan, Huiming Jiang, Xucai Wang, Zizheng Liu, Zheng Wang, Nuo Xu, Wenli Gong, Rui Wu, Dongquan Shi

**Affiliations:** ^1^ Division of Sports Medicine and Adult Reconstructive Surgery Department of Orthopedic Surgery Nanjing Drum Tower Hospital Clinical College of Nanjing University of Chinese Medicine 321 Zhongshan Road Nanjing Jiangsu 210008 China; ^2^ Division of Sports Medicine and Adult Reconstructive Surgery Department of Orthopedic Surgery Nanjing Drum Tower Hospital Affiliated Hospital of Medical School Nanjing University 321 Zhongshan Road Nanjing Jiangsu 210008 China; ^3^ Department of Orthopedics Nanjing Jinling Hospital Affiliated Hospital of Medical School Nanjing University Nanjing 210002 China; ^4^ Department of Orthopedic The Jiangyin Clinical College of Xuzhou Medical University Jiangyin 214400 China; ^5^ Division of Sports Medicine and Adult Reconstructive Surgery Department of Orthopedic Surgery Nanjing Drum Tower Hospital Clinical College of Xuzhou Medical University Xuzhou Medical University Nanjing Jiangsu 221004 China; ^6^ Co‐Innovation Center for Efficient Processing and Utilization of Forest Resources College of Chemical Engineering Nanjing Forestry University Nanjing 210037 China

**Keywords:** ferroptosis, near infrared‐inspired nanoparticle, osteoarthritis, photothermal therapy, TRPV1

## Abstract

Osteoarthritis (OA) is the most common degenerative joint disease worldwide, with the main pathological manifestation of articular cartilage degeneration. It have been investigated that pharmacological activation of transient receptor potential vanilloid 1 (TRPV1) significantly alleviated cartilage degeneration by abolishing chondrocyte ferroptosis. In this work, in view of the thermal activated feature of TRPV1, Citrate‐stabilized gold nanorods (Cit‐AuNRs) is conjugated to TRPV1 monoclonal antibody (Cit‐AuNRs@Anti‐TRPV1) as a photothermal switch for TRPV1 activation in chondrocytes under near infrared (NIR) irradiation. The conjugation of TRPV1 monoclonal antibody barely affect the morphology and physicochemical properties of Cit‐AuNRs. Under NIR irradiation, Cit‐AuNRs@Anti‐TRPV1 exhibited good biocompatibility and flexible photothermal responsiveness. Intra‐articular injection of Cit‐AuNRs@Anti‐TRPV1 followed by NIR irradiation significantly activated TRPV1 and attenuated cartilage degradation by suppressing chondrocytes ferroptosis. The osteophyte formation and subchondral bone sclerosis are remarkably alleviated by NIR‐inspired Cit‐AuNRs@Anti‐TRPV1. Furthermore, the activation of TRPV1 by Cit‐AuNRs@Anti‐TRPV1 evidently improved physical activities and alleviated pain of destabilization of the medial meniscus (DMM)‐induced OA mice. The study reveals Cit‐AuNRs@Anti‐TRPV1 under NIR irradiation protects chondrocytes from ferroptosis and attenuates OA progression, providing a potential therapeutic strategy for the treatment of OA.

## Graphical abstract

Li et al develop a Cit‐AuNRs@Anti‐TRPV1 switch for photothermal activation of TRPV1 signaling for the treatment of osteoarthritis. Cit‐AuNRs@Anti‐TRPV1 has good photothermal responsiveness, and it can rapidly warm up under near‐infrared (NIR) irradiation. By controlling the NIR power and action time, it can realize effective, controllable and targeted activation of TRPV1, thereby suppressing the ferroptosis of chondrocytes to attenuate OA.

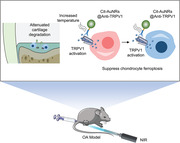

## 1 Introduction

Osteoarthritis (OA) is the most common degenerative joint disease, affecting about 7% of the global population.^[^ ^1^ ^]^ Articular cartilage degeneration has been recognized as the primary pathogenesis of OA.^[^ ^2^, ^3^, ^4^ ^]^ However, effective treatment options to delay osteoarthritic cartilage degeneration are scarcely developed due to the unclear pathogenesis.^[^ ^5^ ^]^

Although chondrocyte ferroptosis has been considered as an important reason of articular cartilage degeneration in OA, effective therapeutic targets resisting ferroptosis are largely unknown.^[^ ^6^ ^]^ The transient receptor potential (TRP) family is a classical device for sensing extracellular signals.^[^ ^7^, ^8^ ^]^ One of them, transient receptor potential vanilloid 1 (TRPV1) is a nociceptive cation channel that can be activated by heat, mechanical stress and capsaicin.^[^ ^9^ ^]^ Previous studies have shown that TRPV1 activation exhibited significant amelioration of OA pain,^[^ ^10^ ^]^ and topical application of capsaicin, a specific agonist of TRPV1, was officially included in knee OA treatment guidelines in 2019.^[^ ^11^ ^]^ We have unveiled molecular and functional features of the ferroptotic chondrocyte cluster in OA and investigated that TRPV1 activation delayed OA progression by suppressing chondrocyte ferroptosis.^[^ ^12^ ^]^ Currently, in clinical trials, the main treatment methods for TRPV1 activation are intra‐articular injection of capsaicin or topical application of capsaicin cream.^[^ ^10^, ^13^ ^]^ Short duration of TRPV1 action, skin damage and unavoidable toxicity due to increasing drug concentration are major disadvantages of both drug delivery methods.^[^ ^13^, ^14^, ^15^ ^]^ Therefore, it remains a challenge to achieve controlled, effective, and targeted activation of TRPV1.

Nanomaterials have been widely used in various biomedical applications such as controlled drug delivery, hyperthermia therapy and optothermal actuation.^[^ ^16^, ^17^, ^18^, ^19^, ^20^, ^21^ ^]^ Multiple nanomaterials‐induced biological effects (e.g., nano‐mechanical, nano‐electric and nano‐thermal effects) can be generated when coupled with external stimulus such as optical, magnetic or acoustic signals.^[^ ^22^, ^23^ ^]^ There is accumulating evidence that the nano‐thermal effects induced by various kinds of nanomaterials can lead to the TRPV1 activation in an effective and controllable manner. Gao et al. used copper sulfide nanoparticles with near infrared (NIR) light irradiation as a photothermal switch for TRPV1 signaling activation in vascular smooth muscle cells to treat atherosclerosis.^[^ ^24^ ^]^ Rosenfeld et al. used the thermal sensitivity of TRPV1 induced by magnetic nanoparticles (MNPs) in remotely applied alternating magnetic fields to drive the rapid release of corticosterone and epinephrine to realize the controllable release of altered hormone levels.^[^ ^25^ ^]^ Moreover, Hescham et al. alleviated Parkinson's‐like symptoms in mice by MNPs‐induced thermal TRPV1 activation, which triggered reversible neuronal firing.^[^ ^26^ ^]^ In view of these, we thought whether chondrocyte ferroptosis prevention and further OA alleviation could be achieved by nanomaterials‐induced thermal TRPV1 activation. Recently, colloidal gold nanorods (AuNRs) with NIR absorption in the range of 600–900 nm has emerged as promising photothermal conversion agents due to their ability to generate localized heating upon laser irradiation, making them a potential tool for the activation of TRPV1. However, untargeted AuNRs may not be suitable for the treatment of OA due to the specific structure of the joint cavity and their relatively short residence time within the OA joint. Therefore, the development of a novel strategy to intelligently target TRPV1‐positive inflammatory macrophages and ferroptotic chondrocytes using AuNRs might be a precise, and pathogenesis‐based OA therapy.

**Scheme 1:**
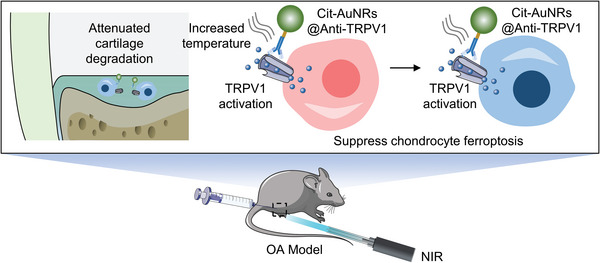
Illustration of Cit‐AuNRs@Anti‐TRPV1 switch for photothermal activation of TRPV1 signaling to attenuate OA.

In this study, we developed a photothermal switch based on the conjugation of AuNRs and TRPV1 monoclonal antibody, providing a therapeutic approach that was expected to attenuate chondrocyte ferroptosis and delay OA progression through the targeted activation of TRPV1 (**Scheme** **1**).

## 2 Results and Discussion

### 2.1 Preparation and Characterization of Citrate‐AuNRs@Anti‐TRPV1

**Figure 1:**
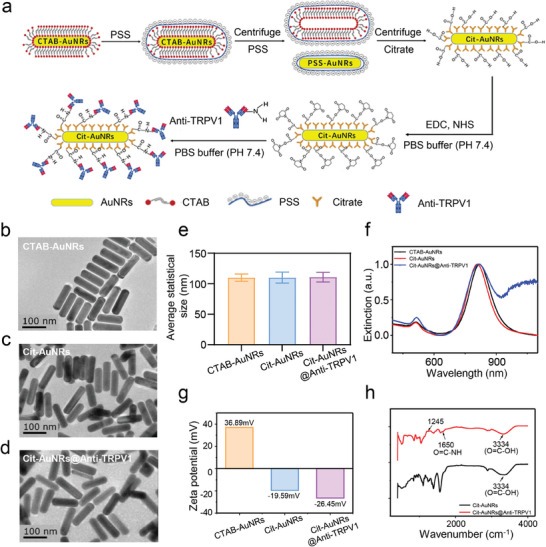
Preparation and characterization of Cit‐AuNRs@Anti‐TRPV1. a) Schematic of preparation of Cit‐AuNRs@Anti‐TRPV1. b,c,d) TEM images, of as‐prepared CTAB‐AuNRs, Cit‐AuNRs and Cit‐AuNRs@Anti‐TRPV1, respectively. e) Statistical average size, f) UV‐vis spectra and g) the zeta (ζ) potential of as‐prepared CTAB‐AuNRs, Cit‐AuNRs and Cit‐AuNRs@Anti‐TRPV1. h) FTIR spectra of Cit‐AuNRs and Cit‐AuNRs@Anti‐TRPV1.

Citrate‐AuNRs (Cit‐AuNRs) were initially synthesized via a PSS‐assisted ligand exchange process from cetyltrimethylammonium bromide (CTAB) stabilized AuNRs. Subsequently, Cit‐AuNRs were employed in conjugation with Anti‐TRPV1 through an amide condensation reaction involving the carboxyl groups on the Cit‐AuNRs and the amino groups on Anti‐TRPV1, resulting in Cit‐AuNRs@Anti‐TRPV1 (**Figure** **1**a). The morphology of the synthesized nanoparticles, including CTAB‐AuNRs, Cit‐AuNRs, and Cit‐AuNRs@Anti‐TRPV1, was characterized through transmission electron microscopy (TEM) imaging (Figure 1b,c,d). The average sizes, determined from TEM images, were found to be 109.1±7.7 nm for CTAB‐AuNRs, 109.6±9.1 nm for Cit‐AuNRs, and 110.5±6.2 nm for Cit‐AuNRs@Anti‐TRPV1, respectively (Figure 1e). These results indicated that the ligand exchange process and subsequent conjugation with Anti‐TRPV1 had no discernible impact on either the morphology or the size of the AuNRs.

To further assess the colloidal stability and surface characteristics of as‐prepared AuNRs, the longitudinal surface plasmon resonance (LSPR) spectrum was employed, and the normalized UV‐vis spectra was presented in Figure 1f. In the case of CTAB‐AuNRs, the absorption peak was observed at 819 nm. While, the UV‐vis spectra of Cit‐AuNRs exhibited a noticeable blue shift to 808 nm due to the changes of surface modification, consistent with our prior research.^[^ ^27^ ^]^ As expected, Cit‐AuNRs@Anti‐TRPV1 exhibited a significant red‐shift in the absorption peak to 815 nm, which could be attributed to the conjugation of Anti‐TRPV1. This observation was in accordance with the findings reported by Tang et al.^[^ ^24^ ^]^ Furthermore, we determined the zeta (ζ) potential of AuNRs using DLS method (Figure 1g). It was evident from the data that CTAB‐AuNRs exhibited a positive ζ potential of 36.89 mV, primarily attributed to the positively charged trimethylammonium group [─N (CH_3_)_3_^+^] present in the CTAB surfactant. Conversely, Cit‐AuNRs displayed a negative ζ potential, measured at −19.59 mV, which can be ascribed to the presence of carboxyl groups on the surface of Cit‐AuNRs. As anticipated, an obvious increase of the absolute value of ζ potential for Cit‐AuNRs@Anti‐TRPV1 (−26.45 mV) could be observed after the conjugation with Anti‐TRPV1. Together with the fact that the Anti‐TRPV1 was negatively charged due to the present of a significant number of amino acid residues with negative charges such as glutamic acid and aspartic acid,^[^ ^24^ ^]^ we confirmed that Anti‐TRPV1 was successfully conjugated onto the surface of Cit‐AuNRs. To further visually verify the conjugation, the chemical structures of Cit‐AuNRs and Cit‐AuNRs@Anti‐TRPV1 were characterized by FTIR. Occurrence of stretching bands (1650 cm^−1^) was associated with the stretching C═O─NH, indicating the formation of amido bond and the successful conjugation of Anti‐TRPV1 (Figure 1h).

### 2.2 Photo‐Thermal Effect of Cit‐AuNRs@Anti‐TRPV1

**Figure 2:**
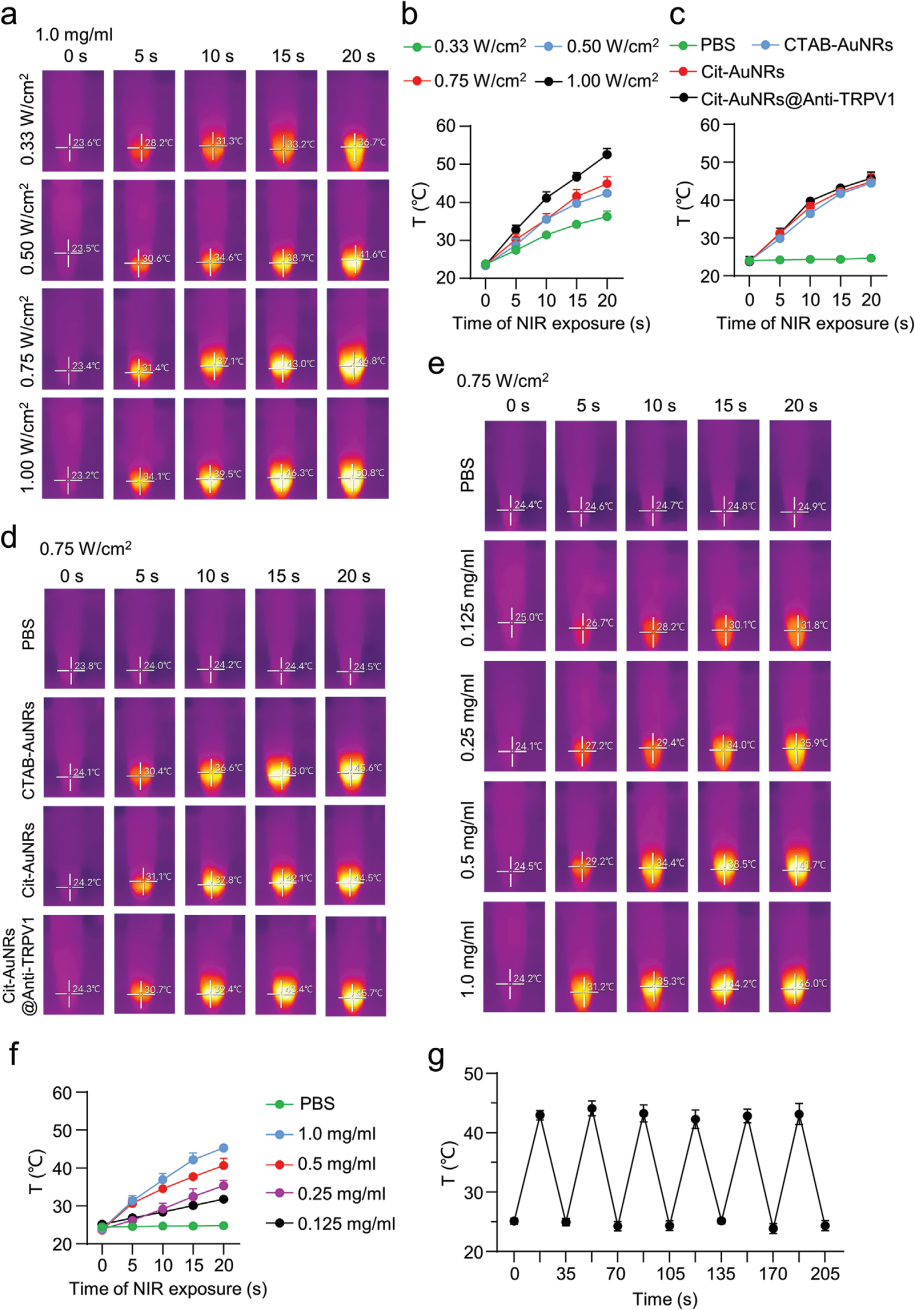
In vitro photothermal effect of Cit‐AuNRs@Anti‐TRPV1. a) Real‐time infrared thermography of Cit‐AuNRs@Anti‐TRPV1 with NIR irradiation at different power levels (1.0 mg ml^−1^, 880 nm for 20 s). b) In vitro temperature change curves of Cit‐AuNRs@Anti‐TRPV1 at different NIR irradiation power levels (1.0 mg ml^−1^, 880 nm for 20 s). c) In vitro temperature change curves of PBS, CTAB‐AuNRs (1.0 mg ml^−1^), Cit‐AuNRs (1.0 mg ml^−1^), Cit‐AuNRs@Anti‐TRPV1 (1.0 mg ml^−1^) with NIR irradiation for 20 s (880 nm, 0.75 W cm^−2^). d) Real‐time infrared thermography of PBS, CTAB‐AuNRs (1.0 mg ml^−1^), Cit‐AuNRs (1.0 mg ml^−1^), Cit‐AuNRs@Anti‐TRPV1 (1.0 mg ml^−1^) with NIR irradiation for 20 s (880 nm, 0.75 W cm^−2^). e) Real‐time infrared thermography of Cit‐AuNRs@Anti‐TRPV1 with NIR irradiation at different concentrations (880 nm, 0.75 W cm^−2^ for 20 s). f) In vitro temperature variation curves of Cit‐AuNRs@Anti‐TRPV1 at different concentrations of NIR irradiation (880 nm, 0.75 W cm^−2^ for 20 s). g) Transient thermal measurements of Cit‐AuNRs@Anti‐TRPV1 (1.0 mg ml^−1^) under repeated cycles of NIR irradiation (880 nm, 0.75 W cm^−2^). Each cycle consisted of 15 s irradiation followed by a 20 s cooling phase. For all graphs, data are expressed as mean ± S.D. of three independent tests.

Firstly, we used NIR to irradiate the 1.5 ml EP tube filled with Cit‐AuNRs@Anti‐TRPV1 in vitro. The NIR source was 5 cm away from the EP tube and the infrared sensor camera was 5 cm away from the EP tube to observe the temperature rise of the material at different power levels. Our results showed that the higher the power of NIR, the better the heating effect of Cit‐AuNRs@Anti‐TRPV1. The temperature of Cit‐AuNRs@Anti‐TRPV1 (1.0 mg ml^−1^, 880 nm, 1.0 W cm^−2^) increased rapidly from 23.2 °C to 50.8 °C within 20 s (**Figure** **2**a). When irradiated with NIR at 0.75 W cm^−2^ for 15 s, Cit‐AuNRs@Anti‐TRPV1 could be warmed up to 43 °C (Figure 2a,b), which is the activation threshold temperature of TRPV1.^[^ ^28^ ^]^ Immediately after that, the photothermal effect of different materials was observed under the same conditions, it was found that CTAB‐AuNRs, Cit‐AuNRs, and Cit‐AuNRs@Anti‐TRPV1 were able to respond to NIR with a good warming effect (Figure 2c,d). Moreover, the photothermal effect was remarkably increased when the concentrations of Cit‐AuNRs@Anti‐TRPV1 increased (Figure 2e,f). Furthermore, after irradiation with NIR at 0.75 W cm^−2^ for 15 s to warm up to ≈43 °C, the EP tube was immediately inserted into the ice and cooled down rapidly (Figure 2g), indicating that Cit‐AuNRs@Anti‐TRPV1 exhibited good temperature regulation capacity.

Strikingly, mouse primary chondrocytes were treated with different concentrations of Cit‐AuNRs@Anti‐TRPV1 for 24 h without NIR irradiation, or irradiated with NIR for 10 cycles (20 s interval with 15 s NIR irradiation at 0.75 W cm^−2^) after fluid exchange to remove the Cit‐AuNRs@Anti‐TRPV1 that was not bound to the cells, or irradiated with NIR of different powers (1.0 mg ml^−1^, 15 s) showed no obvious change in cell viability, suggesting that Cit‐AuNRs@Anti‐TRPV1 under NIR irradiation showed no significant cellular toxicity in vitro (Figure S1, Supporting Information).

### 2.3 The Chondroprotective Effect of Cit‐AuNRs@Anti‐TRPV1 under NIR Irradiation In Vivo

After clarifying the photothermal effect of Cit‐AuNRs@Anti‐TRPV1 in vitro, we sought to demonstrate the potential application of Cit‐AuNRs@Anti‐TRPV1 in vivo. We used 12‐week‐old mice to induce the OA model by performing destabilization of the medial meniscus (DMM) surgery.^[^ ^29^ ^]^ Then, based on the results of in vitro experiments, we intra‐articular injected Cit‐AuNRs@Anti‐TRPV1 (1.0 mg ml^−1^, 8 µl), once a month and irradiated the knee joint with NIR once a week (Figure S2, Supporting Information). In order to ensure that the skin of mice was not injured,^[^ ^30^ ^]^ all mice were performed knee joint depilation and the NIR source was ≈5 cm away from the knee joint. At each treatment, 20 s interval with 15 s NIR irradiation was considered as one cycle, three consecutive cycles were administered each time. After 12 weeks, the knee joints of mice were harvested for histological analysis.^[^ ^31^ ^]^

**Figure 3:**
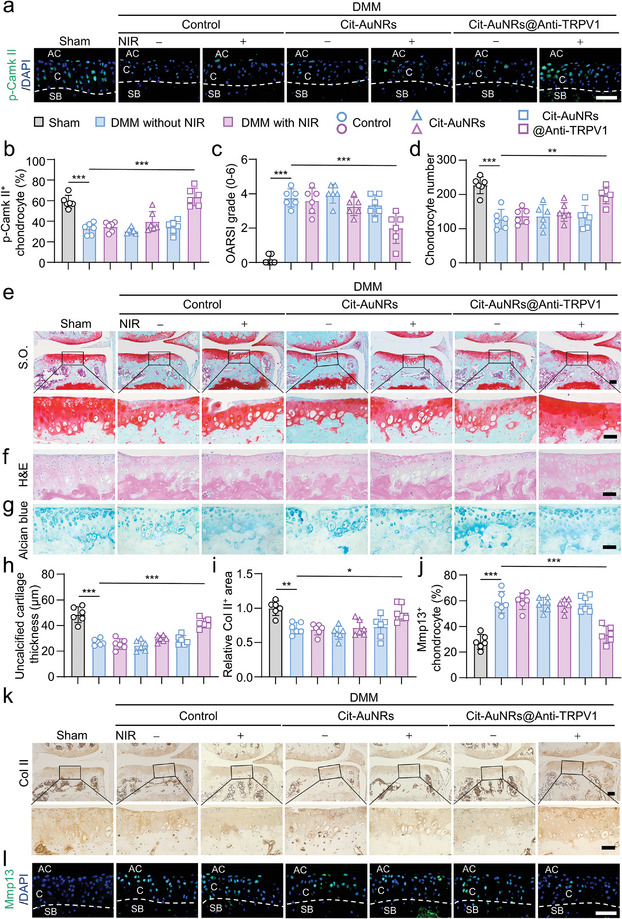
The chondroprotective effect of Cit‐AuNRs@Anti‐TRPV1+NIR in OA. a,b) Immunofluorescence (IF) staining analysis for p‐Camk II expression in the cartilage of mouse by Sham or DMM surgery treated as indicated. AC, articular cavity; C, cartilage; SB, subchondral bone (a) and its quantification (b) (n=6). c) The OARSI score shows the extent of cartilage injury in DMM mice receiving different treatments (n=6). d) The quantification of Safranin‐O/fast green (S.O.) staining (n=6). e) Safranin‐O/fast green (S.O.) staining. f) Hematoxylin & Eosin (H&E) staining of mouse knee. g) Alcian blue staining of mouse knee. h) The quantification of Safranin‐O/fast green (S.O.) staining (n=6). i) The quantification of immunohistochemical (IHC) staining of Col II in the articular cartilage of mouse treated as in (a) (n=6). j) The quantification of IF staining of Mmp13 in the articular cartilage of mouse treated as in (a) (n=6). k) Immunohistochemical (IHC) staining of Col II. (i) Immunofluorescence (IF) staining of Mmp13. Scale bars, 50 µm (a, e (lower panel), f, g, k (lower panel), l), 100 µm (e (upper panel), k (upper panel)). One‐way ANOVA with Tukey's post‐hoc test. Data are shown as mean ± SD. *p< 0.05; **p< 0.01; ***p< 0.001.

First, we used p‐Camk II, a classical downstream protein of TRPV1, as an activation indicator of TRPV1^[^ ^32^, ^33^ ^]^ and found that Cit‐AuNRs@Anti‐TRPV1+NIR treatment was able to significantly reverse the decline of p‐Camk II after DMM surgery, indicating effective activation of TRPV1. Whereas the application of NIR, Cit‐AuNRs or Cit‐AuNRs@Anti‐TRPV1 alone could not activate TRPV1 significantly (**Figure** **3**a,b). Then, we confirmed the role of Cit‐AuNRs@Anti‐TRPV1+NIR in protecting articular cartilage by assessing its integrity. The Safranin‐O/fast green (S.O.) staining, hematoxylin & eosin (H&E) staining, alcian blue staining revealed severe cartilage degradation and an obvious reduction of cartilage thickness after DMM surgery (Figure 3e–g). Surprisingly, after Cit‐AuNRs@Anti‐TRPV1+NIR treatment, the articular cartilage degeneration was evidently alleviated as indicated by decreased Osteoarthritis Research Society International (OARSI) score, increased total chondrocyte count, and improved total cartilage thickness (Figure 3c–h). Further, in the Cit‐AuNRs@Anti‐TRPV1+NIR treatment group, the increase of Col II^+^ area and decrease of Mmp13^+^ chondrocyte percent was observed (Figure 3i–l), suggesting its ability to promote cartilage anabolism and decrease cartilage catabolism. Together, these results reveal that Cit‐AuNRs@Anti‐TRPV1+NIR exerted a chondroprotective role in DMM‐induced OA mice.

Notably, we found Cit‐AuNRs@Anti‐TRPV1 suppresses macrophagic inflammation under NIR irradiation in vivo. H&E staining showed the synovitis score of DMM mice was decreased, and the leukocyte infiltration and synovium thickness were significantly reduced after treatment. More importantly, we observed a prominent decrease in the proportion of CD80^+^ macrophages, a classical type of inflammatory macrophage, after Cit‐AuNRs@Anti‐TRPV1+NIR treatment (Figure S3, Supporting Information). Together, these results suggest that the Cit‐AuNRs@Anti‐TRPV1+NIR treatment simultaneously suppresses chondrocyte degeneration and attenuates synovium inflammation.

### 2.4 Cit‐AuNRs@Anti‐TRPV1+NIR Protects Chondrocyte from Ferroptosis

**Figure 4:**
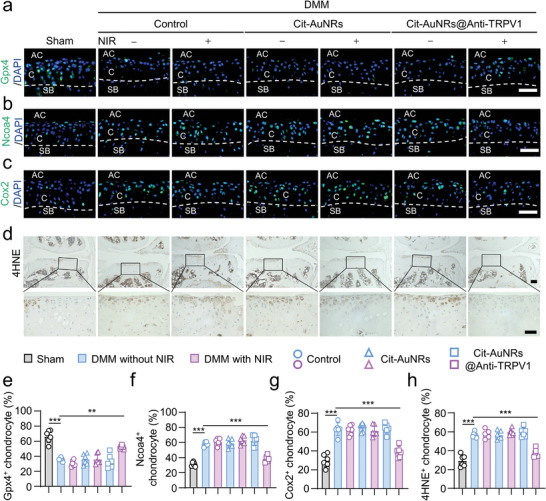
Cit‐AuNRs@Anti‐TRPV1+NIR plays an anti‐ferroptotic role in the mice OA model. a,b,c,d) Representative images of immunofluorescence (IF) staining for Gpx4 (a), Ncoa4 (b), Cox2 (c), and immunohistochemical (IHC) staining for 4‐HNE (d) of mice articular cartilage treated as indicated. AC, articular cavity; C, cartilage; SB, subchondral bone. Scale bars, 50 µm (a, b, c, d (lower panel)), 100 µm (d (upper panel)). e,f,g,h) The quantification of a, b, c, d, respectively (n=6). One‐way ANOVA with Tukey's post‐hoc test. Data are shown as mean ± SD. **p< 0.01; ***p< 0.001.

Ferroptosis is a novel form of programmed cell death characterized by excessive ROS production and iron‐dependent lipid peroxidation.^[^ ^34^, ^35^ ^]^ The regulation of ferroptosis is mainly a game between the its defense system and execution system, in which GPX4 is the core regulatory protein of ferroptosis.^[^ ^36^ ^]^ GPX4 uses its catalytic activity to weaken lipid peroxidation toxicity, maintain membrane lipid bilayer homeostasis, and thus resist ferroptosis.^[^ ^37^ ^]^ In contrast, nuclear receptor coactivator 4 (NCOA4)‐mediated ferritinophagy releases iron stored in ferritin into the unstable labile iron pool, thereby promoting ferroptosis.^[^ ^38^ ^]^ After DMM surgery, the percentage of GPX4^+^ chondrocytes was significantly decreased (**Figure** **4**a,e), the expression of Ncoa4 (Figure 4b,f), ferroptotic marker cyclooxygenase 2 (COX2) (Figure 4c,g) (39), and the lipid peroxidation product 4HNE (Figure 4d,h) (40) were significantly increased. After the treatment of Cit‐AuNRs@Anti‐TRPV1+NIR, the expression of GPX4 was remarkably increased and the expression of Ncoa4, Cox2, and 4HNE were robustly decreased in the articular cartilage, indicating that Cit‐AuNRs@Anti‐TRPV1 under NIR irradiation exerting the chondroprotective effect by suppressing chondrocyte ferroptosis.

### 2.5 Cit‐AuNRs@Anti‐TRPV1 under NIR Irradiation Alleviates Bone Remodeling in DMM Mice

**Figure 5:**
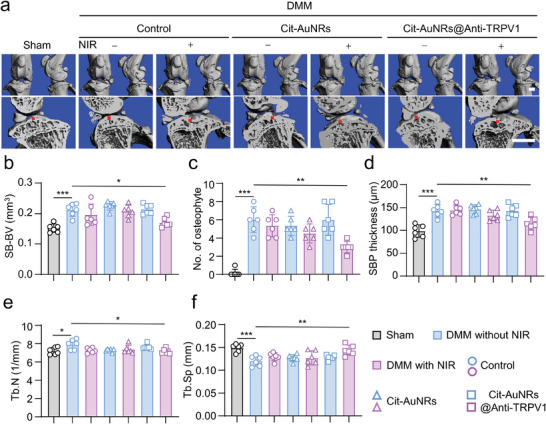
Cit‐AuNRs@Anti‐TRPV1+NIR attenuates bone remodeling in DMM mice. a) 3D images of the mouse knee joint were reconstructed by micro‐computed tomography (micro‐CT) to highlight changes in the femoral and tibial surfaces. The sagittal images of the medial joint compartment show changes in the thickness of the subchondral bone plate (SBP). A red line marks the thickness of SBP. Quantified changes in b) subchondral bone volume (SB‐BV), c) number of osteophytes, d) SBP thickness, e) trabecular Number (Tb.N), f) trabecular separation (Tb.Sp) (n=6). Scale bars, 1mm (a). One‐way ANOVA with Tukey's post‐hoc test. Data are shown as mean ± SD. *p< 0.05; **p< 0.01; ***p< 0.001.

Bone remodeling, including osteophyte formation and subchondral bone sclerosis, is also the main pathological features of OA, which is the subsequent pathogenesis of articular cartilage degradation.^[^ ^41^ ^]^ To evaluate the overall protective effect of Cit‐AuNRs@Anti‐TRPV1 under NIR irradiation on the knee joint, we further analyzed the bone status of the knee joint in mice. 3D reconstruction of the mouse knee joint by micro‐computed tomography (micro‐CT) showed that an increased number of osteophytes and subchondral bone sclerosis after DMM, especially on the medial side, with unsmooth bone surfaces of the tibia and femur and increased subchondral bone volume (**Figure** **5**a–c). These pathological changes were ameliorated in Cit‐AuNRs@Anti‐TRPV1+NIR treated mice. Micro‐CT analysis of the subchondral bone region showed that DMM mice exhibited increased subchondral bone thickness (Figure 5d), trabecular Number (Tb.N) (Figure 5e) and decreased trabecular separation (Tb.Sp) (Figure 5f) compared to mice in the sham group. These OA‐related subchondral abnormalities were resolved by the treatment of Cit‐AuNRs@Anti‐TRPV1 under NIR irradiation. Together, Cit‐AuNRs@Anti‐TRPV1 under NIR irradiation could reverse abnormal remodeling of bone in OA.

### 2.6 Cit‐AuNRs@Anti‐TRPV1 under NIR Irradiation Improves Mice Behavioral Performance

**Figure 6:**
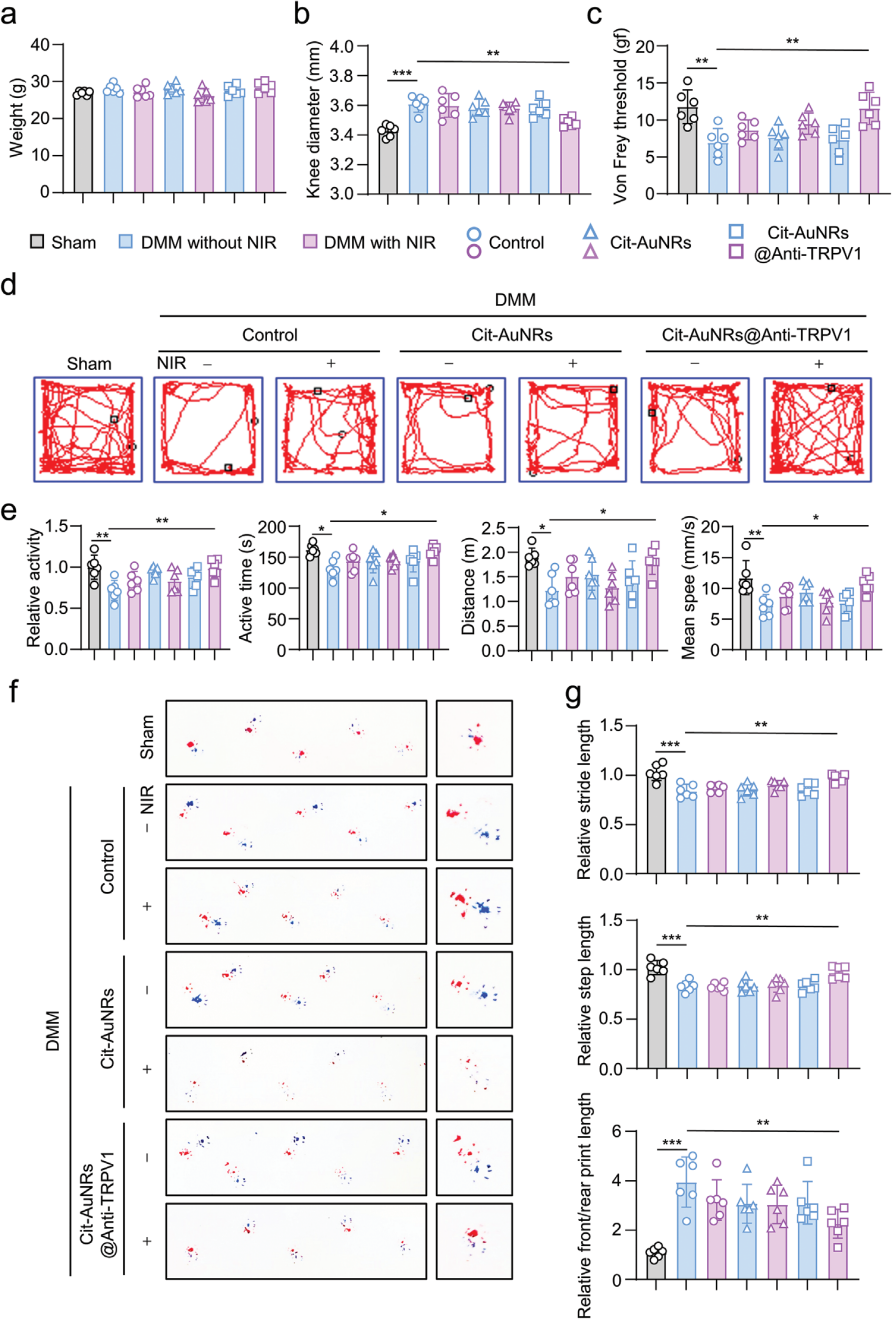
Cit‐AuNRs@Anti‐TRPV1+NIR treatment group improves physical activities and reduces pain in DMM mice. a,b) The weight (a) and the knee diameter (b) of mice treated as indicated (n=6). c) Thresholds of paw contraction were tested using von Frey fibers to reflect mechanical sensitivity (n=6). d) Representative trajectory plots show that the spontaneous activity of mice after DMM surgery decreases in the open field test. Changes in spontaneous activity, including relative activity, active time, distance, and mean speed (e) (n=6). The footprints of the two front paws of the manipulated mice were marked with red ink and the footprints of the two hind paws were marked with blue ink. f) Representative pictures of the footprints of each group. g) Cit‐AuNRs@Anti‐TRPV1+NIR treatment group increased relative stride length, relative step length, and shortened relative front/rear print length in DMM mice (n=6). One‐way ANOVA with Tukey's post‐hoc test. Data are shown as mean ± SD. *p< 0.05; **p< 0.01; ***p< 0.001.

In view of the above findings that Cit‐AuNRs@Anti‐TRPV1 under NIR irradiation exerted an overall protective effect on OA, we further analyzed the physical activities of the mice through behavioral experiments to reflect the knee function. We first evaluate the mice body weight and found no obvious changes among the seven groups (**Figure** **6**a). Whereas Cit‐AuNRs@Anti‐TRPV1 significantly reduced DMM surgery‐induced mice knee swollen under NIR irradiation, as indicated by a decrease in the knee diameter (Figure 6b). We also conducted a series of tests to determine whether Cit‐AuNRs@Anti‐TRPV1+NIR could improve mice behavioral performance in DMM mice.^[^ ^42^ ^]^ First, the results of the von Frey fiber test showed a remarkable decrease in paw withdrawal response thresholds in mice after DMM surgery. Administration of Cit‐AuNRs@Anti‐TRPV1+NIR significantly increased paw withdrawal response thresholds (Figure 6c), indicating reduced pain sensitivity in mice. Second, mice were subjected to an open field test (OFT) to record their spontaneous activity over 3 min (Figure 6d). We observed that the relative activity, active time, distance, and mean speed dramatically declined after DMM surgery relative to the sham group, which was evidently reversed by the Cit‐AuNRs@Anti‐TRPV1+NIR treatment (Figure 6e). Moreover, we further corroborated the pain and gait conditions by footprint experiments,^[^ ^29^ ^]^ and these results indicated that Cit‐AuNRs@Anti‐TRPV1+NIR prominently reduce the pain of DMM mice, which led to the improvement of the stride length and step length, and the shorten of the front/rear print length (Figure 6f,g). In summary, compared with the DMM group, the Cit‐AuNRs@Anti‐TRPV1+NIR treatment group was associated with decreased mechanical pain sensitivity and enhanced spontaneous motion, which significantly improved the physical activities of mice.

### 2.7 Security Validation of Cit‐AuNRs@Anti‐TRPV1+NIR

**Figure 7:**
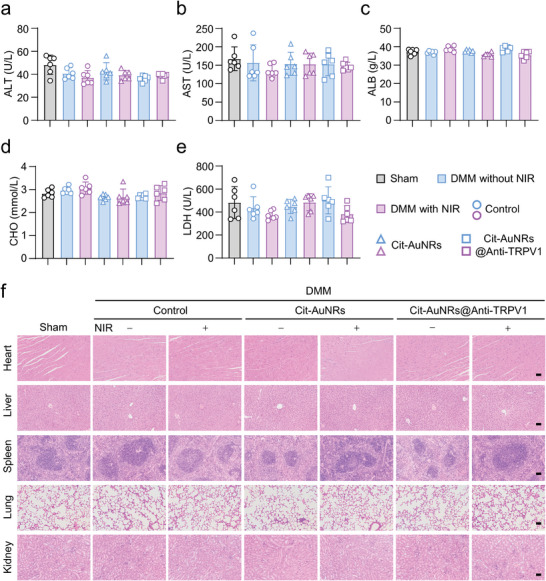
Cit‐AuNRs@Anti‐TRPV1+NIR toxicity evaluation. The serum biochemical analysis for a) alanine aminotransferase (ALT), b) aspartate aminotransferase (AST), c) albumin (ALB), d) cholesterol (CHO), and e) lactate dehydrogenase (LDH) of mice treated as indicated (n=6). f) Hematoxylin & Eosin (H&E) staining of mice heart, liver, spleen, lung, kidney. Scale bars, 50 µm. One‐way ANOVA with Tukey's post‐hoc test. Data are shown as mean ± SD.

Finally, we conducted safety verification of our Cit‐AuNRs@Anti‐TRPV1 under NIR irradiation system. Biochemical tests were carried out on the serum, and there were no significant changes in alanine aminotransferase (ALT) (**Figure** **7**a), aspartate aminotransferase (AST) (Figure 7b), albumin (ALB) (Figure 7c), cholesterol (CHO) (Figure 7d) and lactate dehydrogenase (LDH) (Figure 7e) levels. Additionally, H&E staining was performed on the heart, liver, spleen, lung, and kidney of mice, and discovered no obvious structural and cellular differences among these groups (Figure 7f). These results confirm that Cit‐AuNRs@Anti‐TRPV1+NIR shows no visible toxicity.

## 3 Conclusion

We successfully constructed the Cit‐AuNRs@Anti‐TRPV1 under NIR irradiation system to attenuate cartilage degeneration in OA by protecting chondrocyte from ferroptosis. This study provides a new model for pathogenesis‐targeted precise treatment of OA.

## 4 Experimental Section

### Materials

HAuCl_4_, CTAB and polystyrene sulfonate (PSS), sodium citrate, 1‐ethyl‐3‐(3‐dimethylaminopropyl) carbodiimide (EDC), N‐hydroxysuccinimide (NHS) were purchased from Sigma‐Aldrich Co. Ltd. (MO, USA). Anti‐TRPV1 were obtained from Proteintech (#66 983).

### Preparation of Cit‐AuNRs@Anti‐TRPV1

CTAB‐AuNRs were prepared utilizing a seed‐mediated growth approach, as previously detailed using CTAB as ligands to facilitate stabilization and regulate the synthesis process.^[^ ^43^ ^]^ Cit‐AuNRs were synthesized using a poly (sodium 4‐styrenesulfonate)‐assisted ligand exchange method from as‐prepared CTAB‐AuNRs as previously reported.^[^ ^27^ ^]^ In brief, a solution containing 3 ml of PSS, with a concentration of 10 g L^−1^ within a 5 mM NaCl solution, was combined with 30 ml of CTAB‐AuNRs solution (optical density = 3). The introduction of a modest quantity of NaCl solution was instrumental in facilitating the desired unfolding of the charged PSS chain. The resulting mixture was subjected to incubation at room temperature for 1 h. After that, the solution was subjected to a controlled centrifugation process at 6000 g for 15 min, facilitated the separation of AuNRs from the solution. Following this, the supernatant was gently decanted, and the resulting pellet was meticulously resuspended in 30 ml of deionized water. After repeating this process, the pellet was reconstituted in 30 ml sodium citrate solution (0.1 wt%) and incubated for 12 h. Subsequently, the solution underwent centrifugation at 5500 g for 15 min and the pellet was redissolved with 15 ml sodium citrate solution (0.1 wt%).

Cit‐AuNRs@Anti‐TRPV1 were synthesized using condensation reaction involving the coupling of amino groups of TRPV1 antibody with the carboxyl groups on the surface of Cit‐AuNRs. In brief, Initially, a solution of Cit‐AuNRs (0.05 mg ml^−1^) was prepared, to which 191 µg of EDC and 115 µg of NHS were introduced. This mixture was allowed to react at room temperature for 2 h, facilitating the activation of carboxylate groups on the Cit‐AuNRs’ surface. Subsequently, 10 µg of the TRPV1 antibody was added and mixture was allowed to react at 4 °C for 12 h. After that, the resulting mixture underwent a filtration process using a filter with a molecular weight cutoff (MWCO) of 5 kDa, effectively eliminating the unbound TRPV1 antibody molecules. The resulting product was subjected to multiple washes with deionized water to remove residual reactants and then re‐dispersed in PBS for further use.

### Characterization

Morphological characterization of the AuNRs was performed using TEM (JEM‐2100, Japan). The UV‐vis absorption spectra of as‐parepared AuNRs were recorded with a UV‐vis spectrophotometer (Shimadzu, UV‐3600, Japan). The hydrodynamic size of the AuNRs was measured using Dynamic Light Scattering (Malvern Zetasizer Nano ZS90, UK). The formation of amido bond were confirmed using an FTIR spectrophotometer (Nicolet iS10, Thermo, Waltham, MA, United States).

### Ethics statement

All mouse experiments using mouse were authorized and entirely conducted in accordance with the regulations of the Animal Care and Use Committee of Nanjing Drum Tower Hospital Clinical College of Nanjing University of Chinese Medicine (2020AE01102).

### Cell Counting Kit‐8 (CCK8) Assay

The mouse primary chondrocytes were collected from femoral heads of 3‐days‐old C57BL/6 mice. Cells were cultured in DMEM/F12‐Dulbecco's modified Eagle's medium (Gibco) with the supplementation of 1% penicillin and streptomycin (Gibco) and 10% fetal bovine serum (Gibco) at 37 °C and 5% CO_2_ condition. Chondrocytes were inoculated into 96‐well plates and cultured for 24 h with Cit‐AuNRs@Anti‐TRPV1 induction when the cell density reached 80%, different groups were treated with exchange the culture medium or NIR irradiation. The cell viability was evaluated using a CCK8 assay (#CK04, Dojindo) according to the manufacturer's instructions.

### Animal Study

The animals used in this experiment were all 10‐week‐old male C57BL/6 mice purchased from the Model Animal Research Center of Nanjing University. All animals live in a pathogen‐free rodent housing with a 12 h light and 12 h dark cycle and have free access to food and water. The mice were given the right knee DMM surgically to induce OA model at 12 weeks of age, and the right knee cavity was injected with Cit‐AuNRs or Cit‐AuNRs@Anti‐TRPV1 (8 µl) per month starting at the age of 13 weeks. To confirm the targeting of the material, NIR treatment was chosen to give the day after the injection, at a frequency of once a week (1.0 mg ml^−1^, 880 nm, 0.75 W cm^−2^), 20 s interval with 15 s NIR irradiation was considered as one cycle, three consecutive cycles were administered each time. After 12 weeks of treatment, mice were uniformly sacrificed, then blood, heart, liver, spleen, lung, kidney, and knee joint samples were collected. Furthermore, 12‐week‐old male C57BL/6 mice were randomized to receive:^[^ ^1^ ^]^ Sham surgery (Sham);^[^ ^2^ ^]^ DMM surgery;^[^ ^3^ ^]^ DMM with NIR treatment (880 nm, 0.75 W cm^−2^, three cycles at a time, once a week);^[^ ^4^ ^]^ DMM with Cit‐AuNRs treatment (intra‐articular injection, 1.0 mg ml^−1^, 8 µl per time, once a month);^[^ ^5^ ^]^ DMM with Cit‐AuNRs+NIR (intra‐articular injection, 1.0 mg ml^−1^, 8 µl per time, once a month; 880 nm, 0.75 W cm^−2^, three cycles at a time, once a week);^[^ ^6^ ^]^ DMM with Cit‐AuNRs@Anti‐TRPV1 treatment (intra‐articular injection, 1.0 mg ml^−1^, 8 µl per time, once a month);^[^ ^7^ ^]^ DMM with Cit‐AuNRs@Anti‐TRPV1+NIR (intra‐articular injection, 1.0 mg ml^−1^, 8 µl per time, once a month; 880 nm, 0.75 W cm^−2^, three cycles at a time, once a week), and 8 µl normal saline was given to Sham and DMM groups.

### DMM‐Induced OA Model

Twelve‐week‐old male C57BL/6 mice were anesthetized by inhalation using isoflurane, then the right knee joint was skinned and disinfected with iodophor. Under the stereoscopic microscope, the skin and tissues were dissected layer by layer, the joint capsule was dissected inside the patellar ligament, the soft tissue was bluntly separated by microforceps, and the medial meniscus was exposed. The sharp blade slices off the ligament at the connection between the anterior horn of the medial meniscus and the intercondylar ridge of the tibial plateau. Microforceps were used to confirm that the medial meniscus of the mouse was loose and then the joint capsule and skin were sutured layer by layer and disinfected again. The Sham group was operated identically except that the medial meniscus was not loosened.

### Open Field Test (OFT)

Spontaneous activity and exploratory behavior of the animals were assessed using a tracking system (Zhenghua Technology, China). Each mouse was placed sequentially in an open 50 cm × 50 cm square indoor field without lighting. A camera was used to monitor the mouse's activity trajectory in real‐time within 3 min and to evaluate its relative activity, active time, distance, and mean speed.

### Footprint Experiment

The front and back paws of the mice were dipped in red and blue ink, respectively, and their walking trajectories were recorded using ink blots.^[^ ^44^ ^]^ The red ink marks represent the two front paws and the blue ink marks represent the two hind paws. The mice were allowed to walk freely through a 70 cm long by 20 cm wide track covered with white paper to assess their physical activities and pain in natural walking conditions. And the mice will be acclimated to the environment for one week before the assay. The process was done in a relatively darkroom environment without noise interference, with each mouse tested at least three times, and the ink was safe and non‐toxic.

### Von Frey Fiber Test

Mechanical pain experiments were performed using an electronic von Frey Anesthesiometer (IITC, Woodland Hills, USA). Each mouse was placed in a 4 cm × 3 cm × 7 cm cage with a wire mesh bottom, the mice were allowed to acclimatize on a wire mesh grid for 15 min before the test,^[^ ^45^ ^]^ and the von Frey fiber was applied to the plantar surface of the right hind paw of the mice. By gradually increasing the force applied to the mice paw until the peak detector scores the withdrawal of the mouse paw. The lower the threshold for paw withdrawal, the more sensitive the response to pain. These tests were performed by a researcher who was completely unaware of the experimental animal groupings.

### Immunofluorescence (IF) Staining

Mouse knee sections were dewaxed and hydrated in xylene and graded alcohol, and antigen repair was performed using pepsin for 1 h at 37 °C. After washed by phosphate‐buffered saline (PBS), the slides were blocked with 5% bovine serum albumin (BSA) for 1 h at 37 °C. At last, the slices were incubated overnight (4 °C) with primary antibodies (1:200) of p‐Camk II (#12 716, Cell Signaling Technology), Mmp13 (#GB11247, Servicebio), Gpx4 (#A11243, ABclonal), Cox2 (#12 282, Cell Signaling Technology), and Ncoa4 (#66 849, Cell Signaling Technology). After three washes with TBST, the slices were incubated with fluorescein isothiocyanate (FITC)‐coupled secondary antibody for 1 h at 37 °C. After staining the nucleus by 2‐(4‐Amidinophenyl)−6‐indolecarbamidine dihydrochloride (DAPI), the IF images were acquired using a fluorescence microscope (Zeiss, Germany).

### Immunohistochemical (IHC) Staining

After dewaxing and hydration, the slides were first treated with 3% H_2_O_2_ (protected from light) for 15 min to quench endogenous peroxidase activity. After three washes with PBS, antigen repair was performed with pepsin for 1 h (37 °C). Then, the slices were blocked with 5% BSA for 1 h and final incubated with primary antibodies for col II (#BA0533, Boster), 4HNE (#ab46545, Abcam), CD80 (#66 406, proteintech) overnight (4 °C). Then, the horseradish Peroxidase (HRP)‐ coupled secondary antibody was incubated at room temperature for 1 h. The ultra‐sensitive DAB kit (#1 205 250, Typing) was used for color development, and after staining the nucleus with hematoxylin, the resin was used for sealing.

### Histological Analysis

After micro‐CT analysis, the knee joints of mice were soaked in a 10% EDTA (#1340, Biofroxx) solution for decalcification. The knee joint embedded in the paraffin block was cut into continuous coronal slides (5 µm thick) using a microtome (Thermo, Germany). The slices were detected by Safranin‐O/fast green (S.O.) (#G1371, Solarbio), haematoxylin (H&E) (#C0105M, Beyotime) staining, and Alcian blue (#G1560, Solarbio) staining to observe the integrity and thickness of articular cartilage. The severity of OA was assessed using the OARSI scoring system (0‐6 scale) by blinded observers. The highest OARSI score for each section was recorded and the average of all scores was calculated.

### Micro‐Computed Tomography (Micro‐CT) Analysis

Evaluation of the mouse knee joint by micro‐CT using a Swiss VivaCT 80 scanner (Scanco Medical AG). The scan resolution of the joint was 18.38 µm, and reconstructed 3D images were constructed by Scanco medical software. The cancellous bone was analyzed to obtain trabecular‐related parameters and subchondral bone volume, and the number of bone fragments and subchondral bone thickness was calculated for each knee joint by 3D reconstruction.

### Statistical Analysis

The quantitative data represent at least three independent experiments in which no single mouse or sample was excluded during the analysis. The Levene method was used to verify the homogeneity of variance, and the Shapiro‐Wilk analysis method was used to check whether the data were normally distributed. The unpaired two‐tailed Student's t‐test was used to compare the means values between the two groups. If more than two groups were compared, a one‐way ANOVA followed by Tukey's multiple comparison test was used. All statistical analyses were performed using GraphPad Prism software (version 9.0), and the data were expressed as mean values ±SD. P < 0.05 was regarded as statistically significant.

## Conflict of Interest

The authors declare no conflict of interest.

## Supporting information

Supporting Information

## Data Availability

The data that support the findings of this study are available from the corresponding author upon reasonable request.
