# Supplementary material for: Near Infrared Responsive Gold Nanorods Attenuate Osteoarthritis Progression by Targeting TRPV1
Source: Adv Sci (Weinh). 2024 Feb 15;11(16):2307683. doi: 10.1002/advs.202307683 (PMC11040380; doi:10.1002/advs.202307683)
Supplement: Supplementary file 1 — Supporting Information [file ADVS-11-2307683-s001.pdf]

ADVANCED SCIENCE
Open Access

# Supporting Information

for *Adv. Sci.*, DOI 10.1002/advs.202307683

Near Infrared Responsive Gold Nanorods Attenuate Osteoarthritis Progression by Targeting TRPV1

*Weitong Li, Zhongyang Lv, Peng Wang, Ya Xie, Wei Sun, Hu Guo, Xiaoyu Jin, Yuan Liu, Ruiyang Jiang, Yuxiang Fei, Guihua Tan, Huiming Jiang, Xucai Wang, Zizheng Liu, Zheng Wang, Nuo Xu, Wenli Gong, Rui Wu and Dongquan Shi**

**Supporting information**

**Near Infrared Responsive Gold Nanorods Attenuate Osteoarthritis Progression by Targeting TRPV1**

Li *et al.*

**This file includes:**

Figure S1-S3

Figure S1

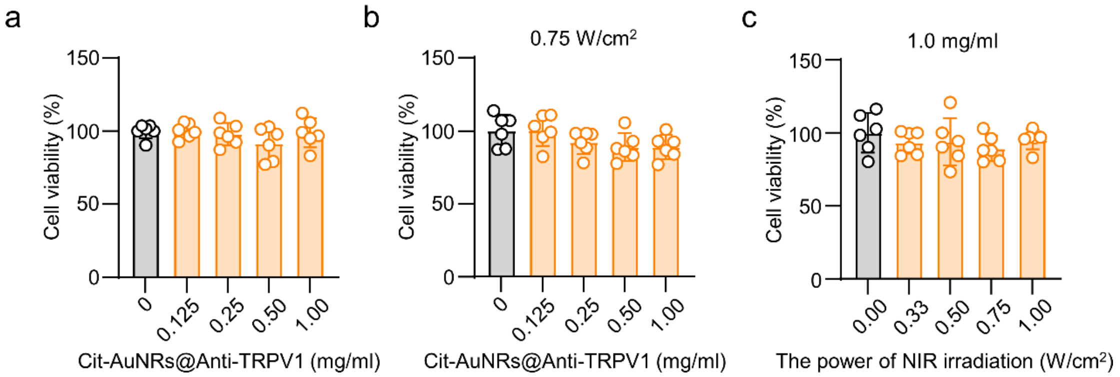


**Figure S1. Cit-AuNRs@Anti-TRPV1 under NIR irradiation was not significantly cytotoxic.** (a) Cell viability of mouse primary chondrocytes treated with various concentrations of Cit-AuNRs@Anti-TRPV1 for 24 h without NIR irradiation (n=6). (b) Cell viability of mouse primary chondrocytes treated with various concentrations of Cit-AuNRs@Anti-TRPV1 under NIR irradiation, 20 s interval with 15 s NIR irradiation at 0.75 W/cm$^2$ was considered as one cycle, lasting 10 cycles (n=6). (c) Cell viability of mouse primary chondrocytes treated with 1.0 mg/ml Cit-AuNRs@Anti-TRPV1 under different power of NIR irradiation for 15 s (n=6). One-way ANOVA with Tukey's post-hoc test. Data are shown as mean ± SD.

Figure S2

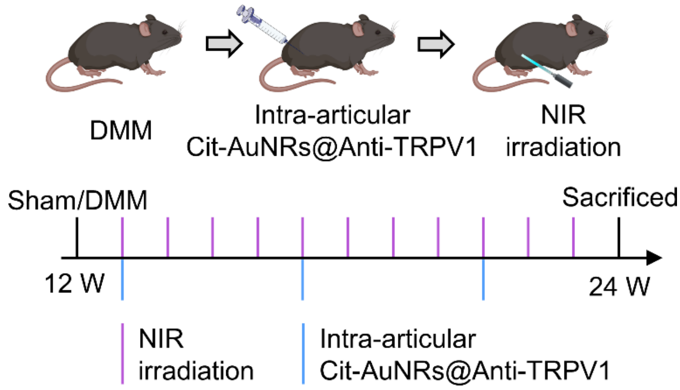


**Figure S2. Timeline for the intra-articular injection of Cit-AuNRs@Anti-TRPV1 and subsequent NIR irradiation.**

Figure S3

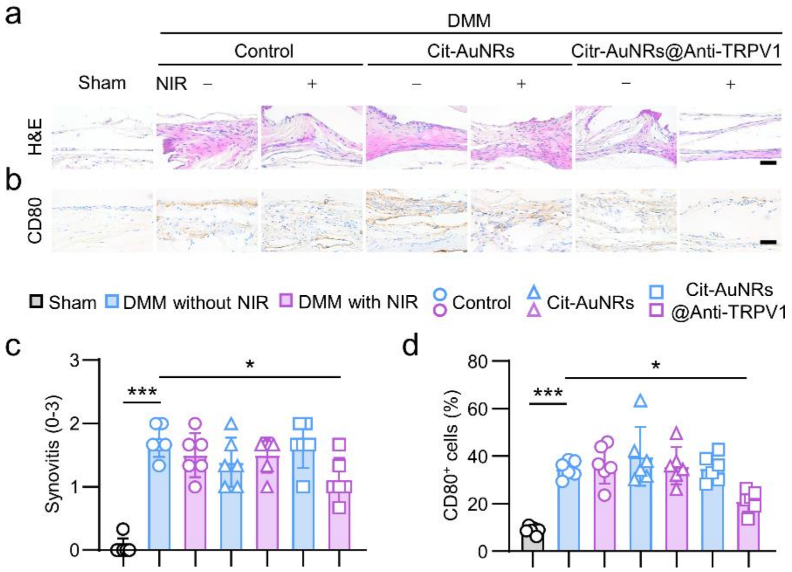


**Figure S3. Cit-AuNRs@Anti-TRPV1 suppresses macrophagic inflammation under NIR irradiation in vivo.** (a, c) Representative images (a) of H&E staining mouse knee sections and the quantified synovitis scores (c) (n=6). (b, d) Representative images (b) of IHC staining and quantification of $CD80^{+}$ cells (d) in the synovium (n=6). Scale bars, 50 μm. One-way ANOVA with Tukey's post-hoc test. Data are shown as mean ± SD. *$p < 0.05$; ***$p < 0.001$.
